# Testing the stress of higher status hypothesis. Variation of occupational stress among physicians and nurses at a German university hospital

**DOI:** 10.1371/journal.pone.0284839

**Published:** 2023-04-25

**Authors:** Sebastian Starystach, Dominik Dauner, Stefan Bär

**Affiliations:** 1 Institute of Medical Sociology and Rehabilitation Science, Charité –University Medicine Berlin, Berlin, Germany; 2 Max-Weber-Institute of Sociology, Heidelberg University, Heidelberg, Germany; North-West University, SOUTH AFRICA

## Abstract

Studies show especially for the UK and the US that physicians experience more occupational stress than nurses. It has also been shown that a higher status within the medical and nursing hierarchy is associated with less occupational stress. Our study’s aim is to examine whether these results also can be found in the context of the German university hospital sector. Thus, we test the stress of higher status hypothesis in and between the occupational groups of nurses and physicians at a German university hospital. Based on two cross-section surveys in the years of 2016 and 2019 this paper compares the perceived level of occupational stress between physicians (n = 588) and nurses (n = 735). Perceived levels of occupational stress–measured via the effort-reward imbalance model and the job demand-control model–are differentiated by status positions within and between both occupational groups. Descriptive as well as inferential statistics (Mann-Whitney U test, Kruskal-Wallis H test) are used to test the stress of higher status hypothesis. Contrary to the stress of higher status hypothesis, our main result is that physicians and nurses perceive similar levels of occupational stress. Furthermore, within each hierarchy the perceived degree of work stress decreases with increasing status for both groups. Our main conclusion is that the stress of higher status hypothesis must be rejected in the German university hospital context and the competing resources of higher status hypothesis must be assumed. The findings can be explained by the unique relationship between physicians and nurses and the role of New Public Management in the German hospital sector.

## Introduction

Even though the COVID-19 pandemic brought about unique challenges and burdensome working conditions for physicians and nurses in the inpatient health care sector [1, 2], however, already for a long time high-strain work can be observed for both occupational groups [3, 4]. Since occupational stress has a measurable impact on patients’ outcome [5], it is important to understand, not only to what extent these occupational groups experience stress, but also to identify the causes of stress, and its relevant dimensions, in order to develop appropriate countermeasures.

Various studies have compared nurses and physicians directly to identify differences in occupational stress between both occupational groups [6–8]. Altogether, the findings support the stress of higher status hypothesis, stating physicians as high-status professionals perceive more occupational stress than nurses [6]. However, a great number of these studies have been conducted in the United States or the United Kingdom [6–8] where nursing has a high status and therefore a high decision autonomy as well as a high level of professionalization. Those characteristics are indicated by tertiary educational qualification and widespread organization in professional associations.

In contrast, German nurses perform a high strain job [3] and perceive more occupational stress than physicians [9] although they are of considerable lower status than their counterparts in the US or the UK. Against this background, the question arises whether the stress of higher status hypothesis can be upheld in the context of the German inpatient healthcare sector.

Based on two cross-section surveys conducted in a university hospital in Germany in the years 2016 and 2019, we test this hypothesis by (a) comparing the extent and structure of occupational stress of nurses and physicians and by (b) examining which influence occupational status has on occupational stress, comparing the effects of status differences between and within both groups.

## Data and methods

### Sample

Two cross-section surveys were conducted in a German university hospital. For each survey, the short questionnaire for workplace analysis [10] in combination with the ERI questionnaire [11] were used. In addition, we used items measuring working conditions in the hospital context that were developed and validated by the authors. The surveys were conducted as part of a legally required occupational group related psychological risk assessment according to the German Occupational Health and Safety Act (‘Gefährdungsbeurteilung psychischer Belastung’, §5 ArbSchG). Due to this, the survey took place at different points in time for nurses and physicians. The first survey which measured the occupational stress and working conditions of nurses was conducted in September 2016. In November 2019 the same instrument was used for the physicians to ensure comparability. In both cases, a paper-based questionnaire was delivered to the employee’s work address via the payroll and a return envelope was enclosed. Via this method, all physicians and nurses who were employed at the university hospital and subject to social security contributions had the possibility to fill out the survey. Since both were employee surveys, there was an information sheet attached, but no written consent form necessary. The survey was approved both by the management through a board resolution and by the staff council.

The survey among nurses had a response rate of 29.4% and among physicians 30.2%. Since both surveys were complete counts, response rates of about 30% can be considered average for the hospital sector in Germany. A non-response analysis based on human resources data was conducted. The results show that both samples can be considered representative for the respective staff structure at the time of data collection with the only exception being that female doctors are slightly overrepresented and male doctors are slightly underrepresented (Table 1).

**Table 1:** Non-response analysis.

	Physicians		Nurses	
	Population	Sample	Population	Sample
	(N = 1717)	(n = 588)	(N = 2496)	(n = 735)
Sex		556		733
• female	42.5% (730)	47.5% (264)	81.9% (2045)	80.6% (591)
• male	57.5% (987)	52.5% (292)	18.1% (451)	19.4% (142)
Employment		561		733
• limited	77.0% (1226)	76.3% (428)	2.2% (55)	3.1% (23)
• permanent	22.1% (348)	23.7% (133)	97.8% (2441)	96.9% (710)
Status group		541	-	-
• residents	49.5% (850)	50.7% (280)		
• specialists	22.7% (389)	22.3% (123)		
• senior physicians	16.2% (278)	19.6% (108)		
• chief physicians	3.0% (52)	5.4% (30)		
Leadership position	-	-		678
• yes			N/A	6.9% (47)
• no			N/A	93.1% (631)

### Operationalization

For analyzing the variation among physicians and nurses, occupational stress was compared by using the well-established effort-reward imbalance model (ERI) by Siegrist [12] and the job demand-control model (JDC) by Karasek [13]. Both models were operationalized as shown in S1 Table.

In addition, twelve dimensions of working conditions were examined to analyze the structure of occupational stress. Using a five-point Likert-scale, all items were coded consistently from burdensome to non-burdensome.

To compare the variation of perceived occupational stress between physicians and nurses, all study participants were categorized in their respective occupational group. To examine the variation of perceived occupational stress differentiated by status, physicians were classified into one of four status groups: residents, specialists, senior physicians, and chief physicians. These terms correspond to the German terms ‘Assistenzarzt’, ‘Facharzt’, ‘Oberarzt’, and ‘Chefarzt’. To reflect differences in status, nurses on the other hand were assigned to either the category ‘in leadership position’ or ‘no leadership position’.

### Statistical analysis

To examine the variation in the degree of occupational stress by status, a distinction was made between physicians and nurses in the first step of the analysis. In the second step, we distinguished between physicians’ status groups within the medical hierarchy. In the third step, we differentiated between status positions within the occupational group of nurses.

For the descriptive statistical data analysis, mean values for twelve specific dimensions of working conditions were calculated. Mean values greater than 3.5 indicate favorable working conditions while mean values lower than 2.5 indicate critical working conditions [14]. These hazardous working conditions are likely to produce occupational stress [15]. The effort-reward ratios were calculated for physicians and nurses in general as well as for each status group within the medical hierarchy. An effort-reward ratio greater than 1.0 indicates the presence of a gratification crisis [11]. The job type according to the job demand-control model was calculated for each status category to differentiate between high and low strain jobs as well as between active and passive jobs [13]. These descriptive parameters were compared between (1) physicians and nurses, (2) between the four status groups within the medical hierarchy, and (3) between status positions within the occupational group of nurses.

To test the stress of higher status hypothesis, the Mann-Whitney U test was used to examine significant differences between physicians and nurses and between status positions within the occupational group of nurses. The Kruskal-Wallis H test followed by the Bonferroni post hoc test were used to examine significant differences between the four status groups within the medical hierarchy. Point-biserial correlations are calculated to measure the effect size. Since none of the dependent variables were normally distributed (p < .001 for all dependent variables in Kolmogorov-Smirnov test and Shapiro-Wilk test), we applied Mann-Whitney U tests and Kruskal-Wallis H tests.

The data analysis was performed with SPSS^®^ 26 and Microsoft^®^ Excel^®^.

## Results

### Descriptive statistics

#### Occupational stress

Table 2 depicts mean values of twelve dimensions of working conditions. Favorable working conditions are light-colored. Critical working conditions that are likely to produce occupational stress are dark-colored.

**Table 2:** Mean values of working conditions differentiated by physicians, nurses, and status groups.

Working Conditions Category	Agency	Versatility	Holistic Nature of Work	Social Support	Cooperation	Work Requirements	Workload	Work Routine	Working Environment	Information and Participation	Career Development	Work-Life Balance
**Physicians Total (n = 557)**	2.64	3.69	3.34	3.53	2.63	3.46	2.01	2.84	2.91	2.56	3.09	2.51
**Residents (n = 260)**	2.38	3.62	3.13	3.58	2.60	3.25	1.99	2.79	2.79	2.57	3.13	2.42
**Specialists (n = 120)**	2.47	3.51	3.27	3.31	2.56	3.52	1.96	2.74	2.64	2.52	2.76	2.50
**Senior Physicians (n = 103)**	2.97	3.87	3.72	3.56	2.67	3.74	1.99	2.93	3.17	2.44	3.16	2.50
**Chief physicians (n = 29)**	3.92	4.29	4.02	4.05	2.86	3.97	2.33	3.28	3.73	3.09	3.70	3.38
**Nurses Total (n = 703)**	2.98	3.56	2.94	3.35	3.18	3.47	1.91	2.56	2.53	2.51	3.08	2.49
**Nurses (no leadership position) (n = 610)**	2.97	3.53	2.91	3.34	3.16	3.45	1.86	2.51	2.47	2.46	3.04	2.46
**Nurses (leadership position) (n = 46)**	3.44	4.04	3.49	3.69	3.59	3.64	2.33	2.95	3.17	3.13	3.82	2.87

Generally, nurses show a higher level of burden than the higher status group of physicians in nine out of twelve dimensions of working conditions. Only in the dimensions ‘agency’, ‘cooperation’, and ‘work requirements’ nurses perceive better working conditions than physicians.

Differentiating the medical profession into status groups, it becomes apparent that physicians of all four status groups perceive high systemic stress due to their workload. Overall, chief physicians have the most favorable working conditions. In comparison to specialists, senior physicians only assess the dimension of ‘information and participation’ as more burdensome. Comparing specialists and residents it is noticeable that specialists consider their working conditions worse than residents in eight out of twelve dimensions.

Against the stress of higher status hypothesis, higher status within the medical hierarchy tends to be associated with less stressful and more favorable working conditions. However, specialists remain the exception. Contrary to the stress of higher status hypothesis, nurses in non-leadership positions regard their working conditions as more burdensome in each of the twelve dimensions.

#### Effort-reward imbalance model

As shown in Table 3, the mean values of the effort-reward ratio of nurses (M = 1.36) and physicians (M = 1.34) indicate the presence of a gratification crisis for both occupational groups on average. There are 77.0% of nurses and 69.4% of physicians with an effort-reward ratio higher than 1.0, indicating a risk of a gratification crisis. In summary, although differences are marginal, the lower status occupational group of nurses does have a higher effort-reward imbalance compared to the high-status occupational group of physicians.

**Table 3:** Effort-reward ratios differentiated by physicians, nurses, and status groups.

	Physicians Total	Residents	Specialists	Senior Physicians	Chief Physicians	Nurses Total	Nurses (no leadership position)	Nurses (in leadership position)
Percentile	n = 533	n = 259	n = 114	n = 95	n = 29	n = 626	n = 539	n = 44
	M = 1.34	M = 1.34	M = 1.49	M = 1.28	M = .96	M = 1.36	M = 1.39	M = 1.01
10	.73	.73	.75	.78	.54	.82	.87	.60
20	.89	.92	.93	.87	.62	.98	1.01	.66
30	1.00	1.02	1.15	.97	.64	1.07	1.09	.81
40	1.12	1.14	1.27	1.05	.74	1.16	1.20	.83
50	1.24	1.27	1.39	1.17	.90	1.28	1.30	.92
60	1.35	1.37	1.50	1.28	.97	1.38	1.41	1.00
70	1.48	1.50	1.65	1.37	1.13	1.52	1.55	1.08
80	1.68	1.69	1.92	1.54	1.35	1.71	1.71	1.24
90	2.05	1.98	2.52	1.96	1.60	1.97	2.00	1.47

Additionally, the mean values of the effort-reward ratio vary widely between status groups within the medical hierarchy. The mean value of the effort-reward ratio of specialists (M = 1.49) is significantly higher than of the other status groups. Moreover, the mean value of the effort-reward ratio of chief physicians is smaller than 1.0 (M = .96), indicating that they are at no risk of a gratification crisis. In summary and contrary to the stress of higher status hypothesis, there is a general trend that the risk of a gratification crisis decreases with increasing status within the medical hierarchy. However, specialists again are the exception, as they are particularly exposed to an effort-reward imbalance.

Finally, the comparison of nurses in non-leadership positions (M = 1.39) and those in leadership positions (M = 1.01) shows extensive differences in the effort-reward ratio. 80.9% of them have an effort-reward ratio of greater than 1.0, indicating a risk for a gratification crisis. In comparison, only 36.4% of nurses in leadership positions have an effort-reward ratio of greater than 1.0. Contrary to the stress of higher status hypothesis we can observe that nurses in a higher status position are exposed to a lower risk of a gratification crisis.

#### Job demand-control model

**Fig 1:**
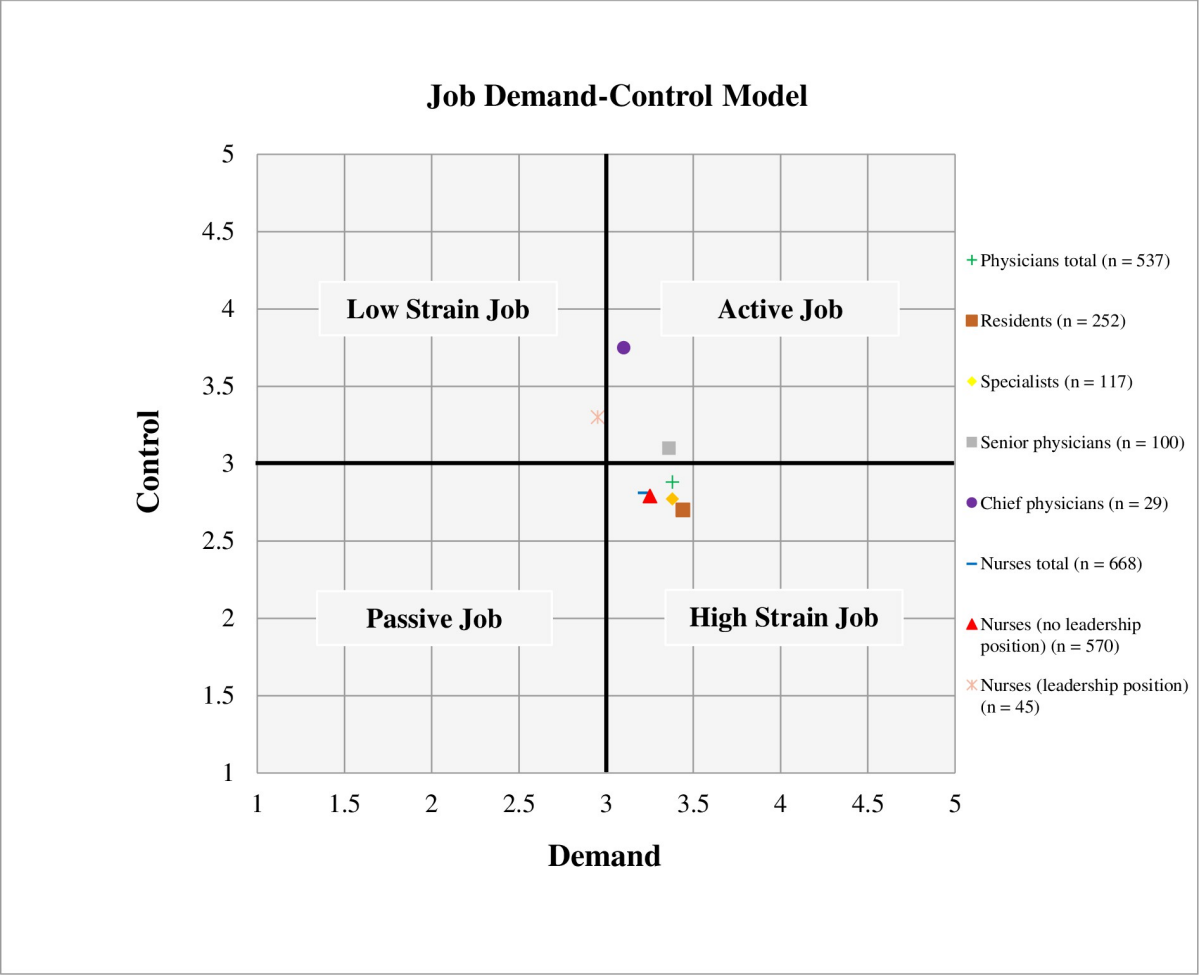
Job type of physicians, nurses, and status groups.

Fig 1 shows that most nurses and physicians work in high strain jobs, that are associated with a high degree of occupational stress. In total, physicians perceive only slightly more demands (M_physicans_ = 3.38; M_nurses_ = 3.22) and slightly more control (M_physicans_ = 2.88; M_nurses_ = 2.81) than nurses. Subsequently, there are no considerable differences between the high-status occupational group of physicians and the lower status occupational group of nurses regarding their job type.

Differentiating the medical profession by status it is apparent that residents and specialists perform a high strain job whereas senior physicians and chief physicians perform an active job. An increase in status within the medical hierarchy is accompanied by a decrease in perceived demands and an increase in perceived control, whereby control increases to a greater extent than demands decrease.

While nurses in non-leadership positions perform on average a high strain job, nurses in leadership positions perform a job type in the transient area from low strain job to active job. Therefore, as status increases, demands decrease (M_non-leadership_ = 3.25; M_leadership_ = 2.95) and control increases (M_non-leadership_ = 2.79; M_leadership_ = 3.30).

### Inferential statistics

In line with the stress of higher status hypothesis, our descriptive results show that physicians perceive a high degree of occupational stress. In contrast however, our results indicate that nurses perceive a high degree of occupational stress as well. Within the medical hierarchy, our results point away from the stress of higher status hypothesis, although specialists represent an exception to this trend. Comparing nurses in leadership positions to nurses in non-leadership positions, our findings also cannot substantiate the stress of higher status hypothesis. In order to generalize these results, it is necessary to examine whether these findings can be also considered statistically significant, which is done in the following.

#### Significant differences between physicians and nurses

First, we examined whether significant differences exist between the descriptive results among physicians and nurses. By using the Mann-Whitney U test, the identified differences in mean values are tested for significance. S2 Table informs whether the null hypothesis is rejected or not with respect to the effort-reward ratio, demands, control as well as the specific dimensions of working conditions. In addition, it indicates the corresponding Pearson correlation coefficient.

The comparison of the effort-reward ratio showed that nurses (M = 1.36) have a marginally higher score than physicians (M = 1.34) (Table 3). Concerning the effort-reward ratio, no statistically significant difference in mean values between nurses and physicians was observed (U = 156570.5, Z = -1.81, p = .071). Therefore, the stress of higher status hypothesis can be rejected with respect to the effort-reward ratio.

Additionally, the comparison of physicians and nurses showed that physicians perceive slightly more demands and slightly more control than nurses (Fig 1). With respect to the job demand-control model, solely in the demand scale a statistically significant difference in mean values between the two occupational groups could be found (U = 156755.5, Z = -5.32, p < .001). Thus, the stress of higher status hypothesis can neither be confirmed nor rejected with respect to the job demand-control model.

Furthermore, our results indicate that nurses perceive more burden than physicians in nine out of twelve dimensions of working conditions (Table 2). There are statistically significant mean differences between the two occupational groups in five out of those nine dimensions. Physicians, by contrast, perceive more burden than nurses only in the dimensions ‘agency’, ‘cooperation’, and ‘work requirements’ (Table 2). In the dimensions ‘agency’ (U = 160061.5, Z = -6.77, p < .001) and ‘cooperation’ (U = 117893.5, Z = -13.32, p < .001), mean differences between the two occupational groups are statistically significant. Thus, concerning the dimensions of working conditions, the stress of higher status hypothesis can neither be confirmed nor rejected.

Overall, contrary to the stress of higher status hypothesis, no general statement can be made as to which of the two occupational groups experiences more occupational stress.

#### Significant differences between status groups within the medical hierarchy

Second, we tested whether significant differences in descriptive results exist among the four status groups within the medical hierarchy. Thus, the Kruskal-Wallis H test was utilized first. S3 Table depicts whether the null hypothesis is rejected or not with respect to the effort-reward ratio, demands, control and the specific dimensions of working conditions.

Regarding the effort-reward ratio (p < .001) as well as demands (p = .012) and control (p < .001), there are statistically significant differences in mean values between the four status groups. Further, there are statistically significant mean differences between the four status groups in all specific dimensions of working conditions except of ‘workload’ (p = .187). Building on these results, a Bonferroni post hoc test using a pairwise comparison must be conducted to determine between which of the four status groups statistically significant mean differences occur (S4 Table).

The descriptive statistical analysis has shown that chief physicians perceive the lowest risk of a gratification crisis (Table 3). As the pairwise comparison between the four status groups within the medical hierarchy reveals, the effort-reward ratios differ significantly between chief physicians and residents (p < .001, r = .233), specialists (p < .001, r = .408), and senior physicians (p = .024, r = .258). Specialists who perceive the greatest effort-reward imbalance with a mean value of 1.49 (Table 3) only show statistically significant mean differences compared to chief physicians (p < .001, r = .408) and senior physicians (p = .024, r = .258), but not compared to residents (p = .194). Thus, the stress of higher status hypothesis can be rejected with respect to the effort-reward ratio.

Regarding the scales of the job demand-control model, our results revealed that with higher status within the medical hierarchy, job demands decrease and control increases. Significant mean differences in the demand scale exist only between chief physicians and residents (p = .008, r = .185). Statistically significant mean differences in the control scale are found in the comparison of all status groups except for specialists and residents (p = 1.00). Therefore, the stress of higher status hypothesis can be rejected with respect to the job demand-control model.

Finally, our results showed that specialists perceive their working conditions worse than residents in eight out of twelve dimensions. Between specialists and residents there are significant mean differences in only three out of twelve dimensions of working conditions. In the dimensions ‘social support’ (p = .032, r = .140) and ‘career development’ (p < .001, r = .204) residents even perceive less burden than specialists (Table 2). Hence, specialists form a status group that is exposed to particularly stressful working conditions and represent an outlier in the general trend, which is that higher status within the medical hierarchy is associated with less occupational stress and less burdensome working conditions.

Once again, contrary to the stress of higher status hypothesis, specialists are an exception to the general trend.

#### Significant differences between nurses in leadership positions and nurses in non-leadership positions

Finally, we examined whether significant differences in the descriptive results among nurses in leadership positions and those in non-leadership positions could be found. Again, the Mann-Whitney U test was used. S5 Table illustrates whether the null hypothesis is rejected or not with respect to the effort-reward ratio, demands, control and the specific dimensions of working conditions. In addition, it depicts the corresponding Pearson correlation coefficient.

Our descriptive results revealed that nurses in non-leadership positions are at higher risk of a gratification crisis (Table 3) and that they perceive more demands and less control (Fig 1) than nurses in leadership positions. Concerning the effort-reward ratio (U = 5369.0, Z = -6.04, p < .001) as well as demands (U = 9466.5, Z = -3.41, p = .001) and control (U = 7285.5, Z = -4.84, p < .001) statistically significant differences in mean values between nurses in non-leadership positions and those in leadership positions were observed. These findings point towards the rejection of the stress of higher status hypothesis.

In addition, nurses in non-leadership positions perceive their working conditions as more burdensome compared to nurses in leadership positions in each of the twelve dimensions (Table 2). There are statistically significant mean differences in eleven out of those twelve dimensions of working conditions. We conclude that nurses in lower status positions regard their level of occupational stress significantly more burdensome than nurses in higher status positions. Therefore, the stress of higher status hypothesis can be rejected with respect to the effort-reward ratio, the job demand-control model as well as specific dimensions of working conditions.

## Discussion

Our results show that both physicians and nurses perform a high strain job and are at a high risk of experiencing a gratification crisis on average. Both occupational groups perceive specific burdensome working conditions and are exposed to occupational stress.

Among physicians, our results are consistent with the assumption of the stress of higher status hypothesis. As members of a profession, physicians have a high degree of autonomy and are authorized to issue instructions to nurses [16]. However, since physician’s professional work is embedded in a bureaucratic organization, this limits the traditionally high degree of autonomy [17]. The buffering effect of autonomy on occupational stress is thus undermined, while professional authority is reinforced by the institutionalized directive power of physicians over nurses, which increases their responsibility and their perceived occupational stress [18, 19]. These results are consistent with the findings of other studies on physicians’ occupational stress in German hospitals. Working conditions and occupational stress of hospital physicians are mainly discussed against the background of increasingly complex cases due to elderly and multimorbid patients as well as constant advances in medical technology [20]. In addition, documentation tasks for physicians are increasing, which translates to growing quantitative work requirements [21]. Increasing efforts lead to an imbalance between efforts and rewards, which in turn results in an increased risk of gratification crises as well [22]. Subsequently, physicians are at higher risk of burnout than other employees in Germany [23].

Contrary to the stress of higher status hypothesis, our results show that nurses, in Germany characterized by a comparatively low status and low degree of professionalization, are also exposed to a high degree of occupational stress. Occupational stress in hospital nursing is discussed within the context of the implementation of New Public Management within the healthcare sector. Here, the shorter length of stay of patients and staff reductions are cited as reasons for increasing work intensification [24, 25]. Work intensification means that breaks from work can be taken less frequently [26], but also that job demands increase [3]. Consequently, hospital nursing in Germany can be characterized as a high-strain job [3]. Further, the relationship between efforts and rewards is out of balance, so that according to a study by Bär and Starystach [3], a large proportion of nurses are at risk of experiencing a gratification crisis. As a result, 37% of nurses in German hospitals are dissatisfied with their work and 17% of nurses plan to leave their profession [27].

Against the background of our findings, it is not possible to draw a general conclusion on which of the two occupational groups perceive more occupational stress. These findings emphasize the unique relationship between both groups in Germany, since various studies from the US or the UK, show that physicians are exposed to more occupational stress than nurses [6–8]. Although our results show that physicians perceive significantly more demands than nurses, nurses assess their concrete working conditions as more stressful than physicians. This raises the question why nurses in Germany are exposed to such high levels of occupational stress. On the one hand, nursing professionalization is progressing comparatively slowly [28], which is why the traditionally hierarchical relationship between the medical profession and nurses persists [29]. This allows physicians to delegate tasks to nurses, resulting in additional workload for nurses and potential reduction in workload for physicians. On the other hand, nursing is a relevant cost factor in hospitals, being the largest occupational group [30]. Due to cost pressures, attempts have been made to systematically rationalize nursing care [24]. Staff reductions led to more work intensification and thus to more occupational stress [21]. By contrast, physicians were able to defend their interests more effectively against these rationalization attempts due to their position as a profession [31].

When comparing the perceived degree of occupational stress among physicians’ status groups within the medical hierarchy, our results indicate that an increase in status is accompanied by an increase in the level of control as well as by a decrease of burdensome working conditions and occupational stress. Few studies have examined status differences in the medical hierarchy regarding occupational health. Albrecht and Giernalczyk [22] state that physicians in higher status positions perceive their occupational health as more favorable than physicians in lower status positions. Consistent with this finding, Kern et al. [32] conclude that residents and specialists perceive less occupational health than senior physicians and chief physicians. Second, when comparing nurses in non-leadership and in leadership positions, it appears that nurses in higher status positions perceive their level of occupational stress to be less burdensome than nurses in lower status positions.

To conclude, our results do not support the stress of higher status hypothesis. To explain this circumstance, we have to consider the hospital organization. First, the hospital’s bureaucratic organization limits physicians’ and nurses’ scope of action [17, 33]. This undermines buffering effects on occupational stress by organizational means. With higher status through advancement in the hierarchy, autonomy can be regained, which in turn serves as a buffering resource and reduces occupational stress [18]. Therefore, at the intra-occupational level, our findings argue in favor of the resources of higher status hypothesis which states that personnel in high-status positions are able to use their job resources in order to buffer occupational stress [34]. At the same time, our results are consistent with Marmot’s [35] considerations on the status syndrome. According to this, personnel in higher positions within the occupational hierarchy has more autonomy than those in lower positions. More autonomy, in turn, is associated with better health outcomes [35].

However, specialists seem to be an exception to this general trend. Although they perceive more occupational stress than senior physicians and chief physicians, at the same time they do not experience less stressful working conditions than residents. That specialists perceive more occupational stress than senior physicians and chief physicians is consistent with the findings of previous research [32]. Furthermore, other studies such as those by Kern et al. [32] as well as Heger and Ritz-Timme [36] show that there are no significant differences between specialists and residents in the perceived degree of occupational stress. One possible explanation for this finding would be that specialists without leadership positions and advanced residents generally have similar tasks [36]. At the same time, residents and specialists have comparatively less decision-making power [32]. Another possible explanation might be that the expected advancement from specialist to senior physician is attainable only for few due to limited positions, thus favoring the risk of gratification crises. Based on these considerations, practical implications can be derived that may help to address this imbalance between efforts and rewards. It would be conceivable to improve the career prospects of specialists by increasing the number of permanent positions below the level of senior physicians.

## Limitations

Regarding the comparison of physicians and nurses, two cross-sectional studies from different years were used. The data was collected as part of a psychological risk assessment according to the German Occupational Health and Safety Act. Both data collection and risk assessment are subject to co-determination, which is why one occupational group was investigated first and then the other. This was not a scientifically based decision in the research design but conditioned by preliminary decisions of the hospital’s board of directors, who wanted to comply with occupational health and safety step by step. Based on the annual reports and preliminary discussions with management and co-determination stakeholders, there were no major changes in this period in organizational structure, management, work organization, patient population, level of care, etc., which is why we assume that comparability is given. Of course, however, we cannot completely rule out the possibility that minor changes, such as staff turnover, could have influenced working conditions and thus limit comparability. Moreover, the results of our case study at a German university hospital cannot be generalized without further ado. In terms of its type, the case studied represents publicly funded university hospitals. Therefore, it is plausible that the results can be generalized to such hospitals with similar structural and contextual conditions. At the same time, this excludes the possibility of generalization to other countries in which nursing is more professionalized. The results presented must be understood against the background of the special, historically developed relationship between the medical profession and nursing in Germany. Another limitation is that we did not differentiate between demographic variables such as age or gender in the presentation of the results. The reason for this is that we assume that status differences are more significant than gender or age differences. Therefore, in order to keep the results explicable in terms of levels of differentiation, we did not include additional gender or age differentiations. Future research may additionally control for these demographic variables and focus on possible interaction effects. Moreover, the questionnaire of our survey does not necessarily measure all dimensions of managerial stress, therefore possible stressful working conditions of chief physicians may not be captured. An indication of this limitation of the instrument is provided by a study by Lindholm [37], which points to the high level of occupational stress among physicians in chief managers’ positions. Nevertheless, we assume that the study is instructive because we were interested in testing the status hypothesis in principle and not so much in the concrete influences of the working conditions on the outcome parameters. For this purpose, it is important, and this is the advantage of our study, that both context and instruments are kept constant in contrast to other studies in which measurements were made in different hospitals with different survey instruments.

## Conclusion

In contrast to studies from countries where nursing has a high status and a high degree of professionalization, our results show that when comparing physicians and nurses at a German university hospital, no clear statement can be made as to which of the two occupational groups is more affected by occupational stress. Instead, a more differentiated perspective concerning the working conditions of physicians and nurses must be taken which focusses on the specific workloads and working conditions of each occupational group. In addition, our study points to the need for an internal differentiation according to status in the analysis of occupational stress for the respective occupational group. We have shown that such a differentiation according to status positions rejects the stress of higher status hypothesis. Therefore, we argue for the assumption of the competing resources of higher status hypothesis in the context of German university hospitals. As a consequence, further research should consider that generalizing findings in international comparisons requires a concept that takes into account the comparability of hierarchies and the respective country-specific institutional context.

## Supporting information

S1 Table Operationalization of the effort-reward imbalance model and the job demand-control model and corresponding cronbach’s α. (DOCX)

S2 Table Mann-Whitney U test for the effort-reward ratio, demands, control, and dimensions of working conditions comparing physicians and nurses. (DOCX)

S3 Table Kruskal-Wallis H test for the effort-reward ratio, demands, control, and dimensions of working conditions comparing status groups within the medical hierarchy. (DOCX)

S4 Table Pairwise comparison of status groups within the medical hierarchy testing statistically significant mean differences for the effort-reward ratio, demands, control, and dimensions of working conditions. (DOCX)

S5 Table Mann-Whitney U test for the effort-reward ratio, demands, control, and dimensions of working conditions comparing nurses in leadership positions and nurses in non-leadership positions. (DOCX)
