# Correction: Testing the stress of higher status hypothesis. Variation of occupational stress among physicians and nurses at a German university hospital

**DOI:** 10.1371/journal.pone.0304538

**Published:** 2024-05-23

**Authors:** Sebastian Starystach, Dominik Dauner, Stefan Bär

## Notice of Republication

An incorrect version of the Data Availability Statement was published in error. This article was republished on May 13, 2024 to correct for this error. Please download this article again to view the correct version.
