# Supplementary material for: Testing the stress of higher status hypothesis. Variation of occupational stress among physicians and nurses at a German university hospital
Source: PLoS One. 2023 Apr 25;18(4):e0284839. doi: 10.1371/journal.pone.0284839 (PMC10128922; doi:10.1371/journal.pone.0284839)
Supplement: S1 Table — (DOCX) [file pone.0284839.s001.docx]

**S1 Table. Operationalization of the effort-reward imbalance model and the job demand-control model and corresponding cronbach’s α.**

| **Effort-Reward Imbalance Model** | | **Job Demand-Control Model** | |
| --- | --- | --- | --- |
| Effort | Reward | Demand | Control |
| - Time pressure - Amount of work - Necessary cooperation with colleagues - Complexity of tasks - Demands on the ability to concentrate - Possibilities to plan work - Interruptions at work - Administrative workload - Burdensome fates of patients - Work with patients | - Recognition by superiors or another important person - Recognition for accomplishments in general - Adequate salary - Appropriate feedback on the quality of one's work - Further training opportunities - Prospect of promotion | - Time pressure - Amount of work - Necessary cooperation with colleagues - Burdensome fates of patients - Work with patients - Administrative workload - Complexity of tasks - Demands on the ability to concentrate | - Possibilities to use knowledge and skills - Possibilities of influencing the allotment of work - Possibilities to plan work - Possibility of determining the sequence of work steps - Holistic nature of work processes - Possibility of evaluation own work performance based on the work result - Interruptions at work - Possibility to contribute ideas |
| α_p_^1^ = .72 | α_p_ = .82 | α_p_ = .67 | α_p_ = .80 |
| α_n_^2^ = .70 | α_n_ = .76 | α_n_ = .68 | α_n_ = .78 |

^1^ α_p_ = Cronbach’s α _physicians‘ sample_

^2^ α_n_ = Cronbach’s α _nurses‘ sample_
