# Supplementary material for: Testing the stress of higher status hypothesis. Variation of occupational stress among physicians and nurses at a German university hospital
Source: PLoS One. 2023 Apr 25;18(4):e0284839. doi: 10.1371/journal.pone.0284839 (PMC10128922; doi:10.1371/journal.pone.0284839)
Supplement: S2 Table — (DOCX) [file pone.0284839.s002.docx]

**S2 Table. Mann-Whitney U test for the effort-reward ratio, demands, control, and dimensions of working conditions comparing physicians and nurses.**

|  | **Mann-Whitney U** | **Z** | **p** | **H_0_** | **r** |
| --- | --- | --- | --- | --- | --- |
| Effort-reward ratio | 156570.5 | -1.81 | .071 | not rejected | - |
| Demand | 156755.5 | -5.32 | < .001 | rejected | .156 |
| Control | 172086.0 | -1.21 | .225 | not rejected | - |
| Agency | 160061.5 | -6.77 | < .001 | rejected | .189 |
| Versatility | 184002.5 | -3.07 | .002 | rejected | .086 |
| Holistic Nature of Work | 153761.5 | -7.73 | < .001 | rejected | .216 |
| Social Support | 179655.5 | -3.89 | < .001 | rejected | .108 |
| Cooperation | 117893.5 | -13.32 | < .001 | rejected | .371 |
| Work Requirements | 203735.0 | -.35 | .720 | not rejected | - |
| Workload | 195286.0 | -1.77 | .077 | not rejected | - |
| Work Routine | 167260.0 | -5.92 | < .001 | rejected | .165 |
| Working Environment | 169133.0 | -5.81 | < .001 | rejected | .161 |
| Information and Participation | 188938.0 | -1.09 | .280 | not rejected | - |
| Career Development | 200328.0 | -.42 | .674 | not rejected | - |
| Work-life Balance | 208721.0 | -.16 | .870 | not rejected | - |
