# Supplementary material for: Testing the stress of higher status hypothesis. Variation of occupational stress among physicians and nurses at a German university hospital
Source: PLoS One. 2023 Apr 25;18(4):e0284839. doi: 10.1371/journal.pone.0284839 (PMC10128922; doi:10.1371/journal.pone.0284839)
Supplement: S3 Table — (DOCX) [file pone.0284839.s003.docx]

**S3 Table. Kruskal-Wallis H test for the effort-reward ratio, demands, control, and dimensions of working conditions comparing status groups within the medical hierarchy.**

|  | **Kruskal-Wallis H** | **df** | **p** | **H_0_** |
| --- | --- | --- | --- | --- |
| Effort-reward ratio | 26.30 | 3 | < .001 | rejected |
| Demand | 10.96 | 3 | .012 | rejected |
| Control | 65.32 | 3 | < .001 | rejected |
| Agency | 75.04 | 3 | < .001 | rejected |
| Versatility | 37.80 | 3 | < .001 | rejected |
| Holistic Nature of Work | 50.99 | 3 | < .001 | rejected |
| Social Support | 17.94 | 3 | < .001 | rejected |
| Cooperation | 8.15 | 3 | .043 | rejected |
| Work Requirements | 33.12 | 3 | < .001 | rejected |
| Workload | 4.80 | 3 | .187 | not rejected |
| Work Routine | 11.79 | 3 | .008 | rejected |
| Working Environment | 27.05 | 3 | < .001 | rejected |
| Information and Participation | 9.48 | 3 | .024 | rejected |
| Career Development | 33.14 | 3 | < .001 | rejected |
| Work-life Balance | 15.79 | 3 | .001 | rejected |
