# Supplementary material for: Testing the stress of higher status hypothesis. Variation of occupational stress among physicians and nurses at a German university hospital
Source: PLoS One. 2023 Apr 25;18(4):e0284839. doi: 10.1371/journal.pone.0284839 (PMC10128922; doi:10.1371/journal.pone.0284839)
Supplement: S4 Table — (DOCX) [file pone.0284839.s004.docx]

**S4 Table. Pairwise comparison of status groups within the medical hierarchy testing statistically significant mean differences for the effort-reward ratio, demands, control, and dimensions of working conditions.**

|  | Chief physicians  –  Residents | Chief physicians  –  Specialists | Chief physicians  –  Senior physicians | Senior physicians  –  Specialists | Senior physicians  –  Residents | Specialists  –  Residents |
| --- | --- | --- | --- | --- | --- | --- |
| Effort-reward ratio | p < .001 ^1^  r = .233 | p < .001  r = .408 | p = .024  r = .258 | p = .022  r = .201 | p = .672  - | p = .194  - |
| Demand | p = .008  r = .185 | p = .078  - | p = .151  - | p = 1.00  - | p = 1.00  - | p = 1.00  - |
| Control | p < .001  r = .414 | p < .001  r = .512 | p = .001  r = .326 | p = .001  r = .249 | p < .001  r = .262 | p = 1.00  - |
| Agency | p < .001  r = .423 | p < .001  r = .547 | p < .001  r = .347 | p < .001  r = .265 | p < .001  r = .264 | p = 1.00  - |
| Versatility | p < .001  r = .274 | p < .001  r = .415 | p = .058  [-] | p = .001  r = .254 | p = .004  r = .173 | p = 1.00  - |
| Holistic Nature of Work | p < .001  r = .301 | p < .001  r = .335 | p = .418  - | p = .003  r = .230 | p < .001  r = .285 | p = .632  - |
| Social Support | p = .050 ^2^  - | p < .001  r = .323 | p = .080  - | p = .146  - | p = 1.00  - | p = .032  r = .140 |
| Cooperation | p = .151  - | p = .070  - | p = 1.00  - | p = .470  - | p = 1.00  - | p = 1.00  - |
| Work Requirements | p < .001  r = .227 | p = .137  - | p = 1.00  - | p = .513  - | p < .001  r = .236 | p = .034  r = .138 |
| Work Routine | p = .016  r = .172 | p = .009  r = .257 | p = .228  - | p = .595  - | p = 1.00  - | p = 1.00  - |
| Working Environment | p = .001  r = .222 | p < .001  r = .351 | p = .232  - | p = .003  r = .228 | p = .029  r = .144 | p = 1.00  - |
| Information and Participation | p = .074  - | p = .045  r = .217 | p = .015  r = .261 | p = 1.00  - | p = 1.00  - | p = 1.00  - |
| Career Development | p = .009  r = .183 | p < .001  r = .422 | p = .032  r = .239 | p = .002  r = .263 | p = 1.00  - | p < .001  r = .204 |
| Work-life Balance | p < .001  r = .227 | p = .004  r = .277 | p = .005  r = .285 | p = 1.00  - | p = 1.00  - | p = 1.00  - |

^1^ Light colored: statistically significant mean differences

^2^ Dark colored: statistically not significant mean differences
