# Supplementary material for: Testing the stress of higher status hypothesis. Variation of occupational stress among physicians and nurses at a German university hospital
Source: PLoS One. 2023 Apr 25;18(4):e0284839. doi: 10.1371/journal.pone.0284839 (PMC10128922; doi:10.1371/journal.pone.0284839)
Supplement: S5 Table — (DOCX) [file pone.0284839.s005.docx]

**S5 Table. Mann-Whitney U test for the effort-reward ratio, demands, control, and dimensions of working conditions comparing nurses in leadership positions and nurses in non-leadership positions.**

|  | Mann-Whitney U | Z | p | H_0_ | r |
| --- | --- | --- | --- | --- | --- |
| Effort-reward ratio | 5369.0 | -6.04 | < .001 | rejected | .250 |
| Demand | 9466.5 | -3.41 | .001 | rejected | .135 |
| Control | 7285.5 | -4,84 | < .001 | rejected | .195 |
| Agency | 9764.0 | -3.67 | < .001 | rejected | .143 |
| Versatility | 8599.0 | -4.62 | < .001 | rejected | .180 |
| Holistic Nature of Work | 9277.5 | -3.94 | < .001 | rejected | .153 |
| Social Support | 10860.5 | -2.88 | .004 | rejected | .112 |
| Cooperation | 9139.0 | -4.22 | < .001 | rejected | .164 |
| Work Requirements | 12394.5 | -1.49 | .136 | not rejected | - |
| Workload | 10529.5 | -3.29 | .001 | rejected | .127 |
| Work Routine | 9423.5 | -3.86 | < .001 | rejected | .150 |
| Working Environment | 9812.0 | -3.55 | < .001 | rejected | .138 |
| Information and Participation | 8020.5 | -4.93 | < .001 | rejected | .193 |
| Career Development | 7528.0 | -5.39 | < .001 | rejected | .209 |
| Work-life Balance | 11588.0 | -2.43 | .015 | rejected | .094 |
